# Supplementary material for: Activated polymorphonuclear derived extracellular vesicles are potential biomarkers of periprosthetic joint infection
Source: PLoS One. 2022 May 9;17(5):e0268076. doi: 10.1371/journal.pone.0268076 (PMC9084519; doi:10.1371/journal.pone.0268076)
Supplement: Attachment — Submitted filename: response_to_reviewers.docx [file pone.0268076.s002.docx]

Response to reviewers

Response to reviewer #1:

1) *In the Introduction when discussing EVs, the authors should cite a paper that is related, Specifically, they should cite KR Genschmer el al. Cell 176, 113-126, 2019. This paper describes exosome-sized EVs from activated PMNs that are found in COPD and likely cause disease.*

Answer: thank you for your recommendation. We inserted the mentioned article.

2) *In Figure 2 there are numerous peaks between 250 and 450 nm and they should be described as such. It seems erroneous to conclude that there are only peaks at 300 and 400 nm.*

Answer: thank you for the remark. Re-evaluation of the Fig. 2 was made and the corresponding text was also corrected as follows: „The septic group showed a shift towards the larger sizes and formed multiple peaks between 300 nm and 400 nm range„.

3) *On page 13 lines 256 and 261, Figure 2A and B are reversed from the order found in the Figure legend.*

Answer: corrected

4) *As presented Figure 3 is not particularly helpful. It would be improved by adding arrows to indicate what is being described in the text such as "dense cargo", "thin regular membrane", etc.*

Answer: arrows and circles were inserted to indicate the characteristic parts of the image.

5) *There are numerous spelling errors that should be corrected.*

Answer: we have corrected spelling mistakes.

Response to reviewer #2:

*In this study Sallai et al investigated extracellular vesicles (EV) from synovial fluid of patients with prosthetic joint inflammation and aseptic loosening. Their findings are valuable for researchers in this field and possibly for clinicians as well on the long run.*

*Although the methods used are of high scientific value and quality, but the relatively low number of patients enrolled and compared is a weakness.*

Answer: We agree with the reviewer, the number of patients is relatively low, this is why we have also highlighted it in the limitations section. However, due to the complexity of our investigation other authors usually work with similar sample sizes. (e.g. Rühwald et al., J Clin Med. 2020;9(2).)

*The introduction section is clear and well written, although “entailing serious impact ... on health care providers”, “analysing and comparison” could be better phrased.*

Answer: corrected

*Reference 8 is a review about miRNA in lung cancer, please replace it with a study that supports the role of EV in cancer.*

Answer: due to the comment of reviewer #1 we inserted one citation before the reference 8. The reference number 8 changed number 9. This reference was replaced with another latest article: Osti D, et al. Clinical significance of extracellular vesicles in plasma from glioblastoma patients. Clinical Cancer Research. 2019;

The aims of the study are clearly stated. The methods used are adequate.

The study cohort consisted of 17 patients, who were divided into two subgroups, which seem quite low. The methods used in this basic research study could justify this, and this weakness was addressed in the limitations section. How does this number compare to the studies cited in the introduction (Ref 1-11)?

Answer: To our knowledge there is only one paper published in the literature dealing with EVs in the synovial fluid of PJI patients. This paper by Rühwald et al. (reference number 27) evaluates the results of 23 patients, which is less, the our cohort of 34 patients.

*The authors state, that not all samples have been analysed by all methods, due low volume of obtained synovial fluid. How did the authors decide which sample would be analysed by a certain method? How were patient numbers in these groups decided, they seem rather random.*

Answer: Samples were selected at random. Due to the low sample number power analysis was not performed.

*Did the authors considered using less investigative methods, which would have allowed them to analyse more samples in those one or two methods?*

Answer: In order to use an up-to-date and widely accepted EV preparation method the MISEV 2018 guidelines were followed throughout our study. This consensus protocol uses different investigative methods which are required to evaluate all the properties of EVs. In the absence of all the required investigations our results were hard to interpret.

*Patient in the acute septic group never received antibiotics, they were immediately scheduled for revision surgery?*

Answer: yes, they never received antibiotics before the surgical treatment. They were scheduled immediately for revision surgery and they received the first dose of antibiotic only after surgical approach and sample harvesting.

*Table 1 shows patient groups, and some inflammation markers. Only these markers were used to assign patients to groups? Or the scoring system described in Ref 12, mentioned in line 147 was used? If so, then what were the exact scores for these groups?*

Answer: The International Consensus Meeting on Periprosthetic Joint Infection criteria were used in our study; their scoring system was also calculated. The missing data is added now to Table 1.

*I recommend not using empty rows for dividing the table, and in the fifth cell of the last column there is an “x” instead of “4”.*

Answer: thank you, corrected.

*Throughout the study patients were divided into three groups and their data was analysed that way. However, for Nanoparticle tracking analysis they were divided into two groups? What is the explanation for this?*

Answer: Different methods were used to evaluate the size distribution of EVs, first Dynamic Light Scatter (DLS), then, according to the international trends, we switched to Nanoparticle Tracking Analysis (NTA). As it happened with other investigative methods, we did not have enough samples to run all the tests in every sample. We have decided to use NTA in the final version of the manuscript to follow the recent MISEV guidelines.

*Larger particles with different morphological properties were observed in the septic group, however these alterations were not statistically analysed, or proved to be significant. This should be noted in the Discussion section.*

Answer: thank you for the recommendation, we noted this in the Discussion section.

*The finding of larger particles in the septic group contradicts Ref 26, as noted by the authors. Any possible explanation for these conflicting results?*

Answer: Rüvald et al. (ref. mentioned above) used a different EV isolation protocol. They used ultracentrifugation 100,000x g for 2 hours. This isolation protocol results in other – smaller – population of vesicles. This explanation has been added to the discussion section.

*Has there been any study investigating CD177 expressing cells in PJI, to which the study results could be compared? The summary is clean and concise, however discussing clinical application seems a bit far reaching to me.*

Answer: NB1 (CD177) expression on the surface of neutrophils is facilitating the trans-endothelial migration of the cell. To our best knowledge there is only one research *(Li, R., Song, L., Quan, Q., Liu, M. W., et al. (2021). Detecting Periprosthetic Joint Infection by Using Mass Spectrometry. JBJS, 103(20), 1917-1926.)* mentioning investigation of CD177 expressing cells in PJI diagnostics. This research investigated periprosthetic synovial fluid via Mass Spectrometry (MS) and found higher expression of polymorphonuclear leukocyte serine protease 3 (PRTN3) which is involved in the selective recruitment of CD177 positive neutrophils and apoptosis of neutrophils. They found also higher expression of lactoferrin and leukocyte serin protease 3. In our MS analysis we found higher expression of lactoferrin and some serine protease also.

*The Funding statement is not clear for me. Data in parenthesis in the details of the ENDO-Verein support, or a separate funding?*

Answer: two separate funding. Corrected.

Funding

This work was supported by the ENDO-Verein Hamburg, Germany. The funders had no role in study design, data collection and analysis, decision to publish, or preparation of the manuscript.

The study won a tender EFOP-3.6.3-VEKOP-16-2017-00009 which we used to purchase equipment.

----------------------------------------

Other remarks:

Alpha-2-HS-glycoprotein is usually abbreviated as AHSG, and not AHSGP. - corrected

Typos, grammatical errors: line 116, 142, 322, 324, 406, 408 - corrected
